# Supplementary material for: Tokenized and continuous embedding compressions of protein sequence and structure
Source: Patterns (N Y). 2025 Jun 13;6(6):101289. doi: 10.1016/j.patter.2025.101289 (PMC12191763; doi:10.1016/j.patter.2025.101289)
Supplement: Document S1. Figures S1 and S2, Tables S1–S4, and Data S1 [file mmc1.pdf]

Patterns, Volume *6*

Supplemental information

Tokenized and continuous embedding compressions of protein sequence and structure

Amy X. Lu, Wilson Yan, Kevin K. Yang, Vladimir Gligorijevic, Kyunghyun Cho, Pieter Abbeel, Richard Bonneau, and Nathan C. Frey

Tokenized and Continuous Embedding Compressions of Protein Sequence and Structure
Amy X. Lu[1,2,*,**], Wilson Yan[1], Kevin K. Yang[3], Vladimir Gligorijevic[2], Kyunghyun Cho[2,4], Pieter Abbeel[1], Richard Bonneau[2], and Nathan C. Frey[3]

[1]University of California, Berkeley
[2]Prescient Design, Genentech
[3]Microsoft Research
[4]New York University
[*]Correspondence: amyxlu@berkeley.edu
[**]Lead Contact: amyxlu@berkeley.edu

Supplemental information

Data S1. Code snippet for obtaining the embedding space used in this work.

Table S1. FSQ codebook levels used to obtain the implicit codebook size in Figure 4.

Table S2. Compression results on sequence-only datasets.

Table S3. Benchmarks on function and localization.

Table S4. Benchmarks on structural and contact tasks.

Figure S1. TSNE of embeddings after and before compression, colored by the "Architecture" level of the CATH classification hierarchy.

Figure S2. Exploring effects of corrupting the token representations on the structure space.

Extracting Embedding from ESMFold

We provide a code sketch of how to obtain the embedding space used in this work, based on modified and annotated source code from ESMFold[1]. Note that `self.esm_s_combine` and `self.esm_s_mlp` are both trained end-to-end with loss objectives from the original ESMFold paper; these weights add more structural awareness to the embedding space which we examine in this work than the original ESM2 3B embedding.

```
# self.esm_s_combine is a learnable weight
self.esm_s_combine = nn.Parameter(torch.zeros(self.esm.num_layers + 1))

# Forward pass through ESM2 language model.
# ‘esm_s‘ is a stack of representations from all layer outputs
# esm_s = torch.stack(
#   [v for _, v in sorted(res["representations"].items())], dim=2
# )
# Typically, when working with ESM2 LM embeddings, one would use
# embeddings from ‘res["representations"][final_layer_number]
esm_s, esm_z = self._compute_language_model_representations(esmaa)

# weigh representations from different layers
esm_s = (self.esm_s_combine.softmax(0).unsqueeze(0) @ esm_s).squeeze(2)

# Simple MLP for manipulating output representation
s_s_0 = self.esm_s_mlp(esm_s)

# Pairwise input is initialized to zero:
s_z_0 = s_s_0.new_zeros(B, L, L, self.cfg.trunk.pairwise_state_dim)

# Pointwise add embedding of amino acid
s_s_0 += self.embedding(aa)

########
# Use this representation for CHEAP experiments:
# return s_s_0
########

# In ESMFold code, this embedding is then used as input
# to the structure trunk. We also use this to
# reconstruct structure from the CHEAP embedding.

structure: dict = self.trunk(
    s_s_0, s_z_0, aa, residx, mask, no_recycles=num_recycles
)
```

Data S1: **Code snippet for obtaining CHEAP embeddings from ESMFold.** Adapted code snippet specifying the layer used to define the ESMFold latent space. We use the layer just prior to the structure module input, due to our observation that the pairwise input into the Structure Module is initialized as zeros (Main Text Figure 1).

FSQ codebook

To obtain the codebook sizes used in Figure 4, we use the same FSQ levels as in the original FSQ paper[2].

**Target Size** $\lvert C \rvert$	2^8	2^{10}	2^{12}	2^{14}	2^{16}
**FSQ levels**	[8, 6, 5]	[8, 5, 5, 5]	[7, 5, 5, 5, 5]	[8, 8, 8, 6, 5]	[8, 8, 8, 5, 5, 5]

Table S1: **FSQ levels used to define codebook sizes.** Mirroring the original FSQ paper, we use the same FSQ levels to define the codebook size $\lvert C \rvert$ in Figure 4.

Compression Results on Sequence-Only Datasets

	Method	Dimensions		Structure metrics				Sequence metrics
		Codebook	Dim.	TMScore		RMSD		Sequence recovery
				Mean	Median	Mean	Median	Mean
	Cont.	-	256 x 32	0.939	0.983	1.740	0.740	0.755
	Cont.	-	256 x 64	0.959	0.991	1.060	0.601	0.756
	Cont.	-	512 x 8	0.883	0.961	2.707	1.424	0.753
Pfam	FSQ	[8,8,8,6,5]	-	0.711	0.716	5.523	4.742	0.555
Pfam	FSQ	[16,16,16,16]	-	0.706	0.698	5.673	4.673	0.538
Pfam	Cont.	-	128 x 8	0.760	0.819	5.273	3.739	0.622
Pfam	Cont.		256 x 4	0.564	0.539	10.203	9.927	0.471
Pfam	Cont.	-	256 x 8	0.728	0.786	6.062	4.320	0.471
Pfam	Cont.	-	256 x 32	0.826	0.947	3.740	1.561	0.812
Pfam	Cont.	-	256 x 64	0.972	0.975	1.855	0.837	0.975
Pfam	Cont.		512 x 4	0.552	0.522	10.264	10.065	0.438
Pfam	Cont.	-	512 x 8	0.850	0.925	3.274	1.963	0.960
CATH	FSQ	[8,8,8,6,5]	-	0.853	0.861	3.670	3.668	0.820
CATH	FSQ	[16,16,16,16]	-	0.875	0.909	3.208	2.900	0.937
CATH	Cont.	-	256 x 8	0.856	0.918	4.170	2.647	0.991
CATH	Cont.	-	256 x 32	0.931	0.971	2.075	1.185	1.000
CATH	Cont.	-	256 x 64	0.928	0.992	1.769	0.489	1.000

Table S2: **Compression results on sequence-only datasets.** TMScore is calculated with respect to the ESMFold predicted structure, rather than the ground truth structure.

Comparison with Other Embedding Methods on PEER Benchmark

Extending Figure 5, we also include comparisons to other reported performances in Xu et al.[3]:

	# Dimensions	Flu ↑	Sta ↑	β-lac ↑	Sol ↑	Sub ↑	Bin ↑
DDE	400	0.64	0.65	**0.62**	0.60	0.49	0.77
Moran	240	0.40	0.32	0.38	0.58	0.31	0.56
LSTM	640	0.49	0.53	0.14	0.70	0.63	0.88
Transformer	512	0.64	0.65	0.26	0.70	0.56	0.76
CNN	21	**0.68**	0.64	0.78	0.64	0.59	0.83
ResNet	512	0.64	0.13	0.15	0.67	0.52	0.79
ProtBert	1024	0.34	0.70	**0.62**	0.59	0.59	0.82
ESM-1b	1280	0.43	**0.75**	0.53	0.67	**0.80**	0.92
**CHEAP (ours)**	4	0.14	0.40	0.13	0.60	0.33	0.68
	8	0.22	0.44	0.17	0.64	0.45	0.74
	16	0.27	0.55	0.23	0.65	0.54	0.84
	32	0.28	0.56	0.28	0.67	0.57	0.87
	64	0.31	0.56	0.28	0.69	0.62	0.90
	128	0.41	0.58	0.38	0.70	0.68	0.90
	256	0.47	0.60	0.41	0.71	0.72	0.92
	512	0.51	0.63	0.36	**0.72**	0.74	0.93
No compression	1024	0.52	0.64	0.45	**0.72**	0.76	**0.94**

Table S3: **Benchmarks on function and localization.** Full results for Main Text Figure 6 on the PEER benchmark[3], with baseline results directly taken from the PEER paper.

	# Dimensions	Cont	Fold	SSP	Yst
DDE	400	–	0.10	–	0.56
Moran	240	–	0.07	–	0.53
LSTM	640	0.26	0.08	0.69	0.54
Transformer	512	0.18	0.09	0.60	0.54
CNN	21	0.10	0.11	0.66	0.55
ResNet	512	0.20	0.09	0.70	0.49
ProtBert	1024	0.40	0.11	0.82	0.54
ESM-1b	1280	0.46	0.30	0.83	**0.66**
**CHEAP (ours)**	8	0.28	0.15	0.82	0.45
	64	**0.42**	0.45	0.85	0.48
	128	0.38	0.47	0.85	0.51
	256	0.23	0.50	0.85	0.51
	512	0.37	**0.53**	**0.86**	0.46

Table S4: **Benchmarks on structural and contact tasks.** Full results for Main Text Figure 6 on the PEER benchmark, with baseline results directly taken from the PEER paper.

Compressed Representation TSNE Plots

TSNE of embeddings after and before compression, colored by the "Architecture" level of the CATH classification hierarchy. Qualitative examination shows that despite $128\times$ compression, fold and topology information is still large preserved.

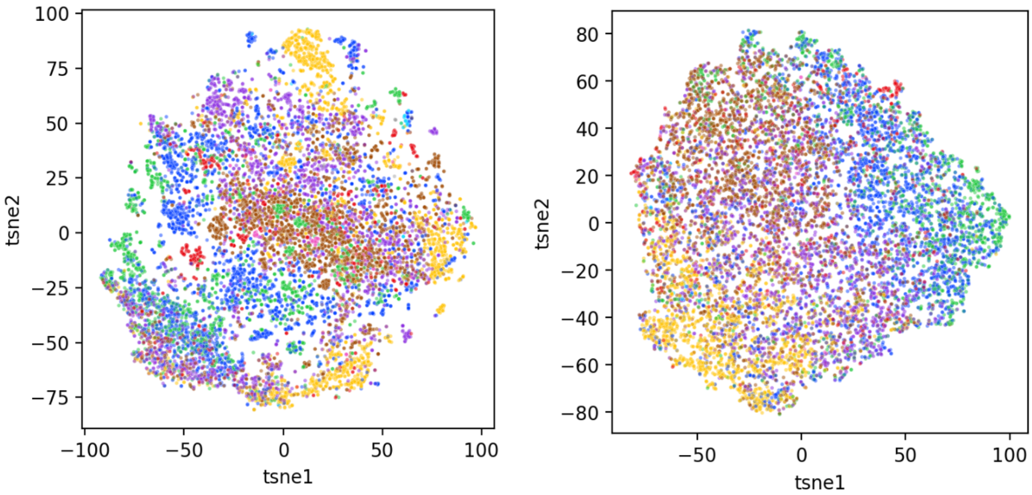

Figure S1: **TSNE of embeddings after and before compression.** Colored by the "Architecture" level of the CATH classification hierarchy.

Token Masking on Structure Reconstruction

Mirroring the analysis in Figure 7, we also examine the effect of "disturbing" the tokens on the output structure (Figure 4C), as a sanity check of how tokens encode structural information. The examined model uses FSQ levels $[16, 16, 16, 16]$, and has an implied codebook size of $C = 16^4 = 65536$. Since there is no `[MASK]` token in the vocabulary, we set different pre-quantization embeddings to 0. Generally, changing a subset of the tokens changes the structure, though since the codebook representations are not evenly spread through out, setting the first channels to zero has less of a detrimental effect.

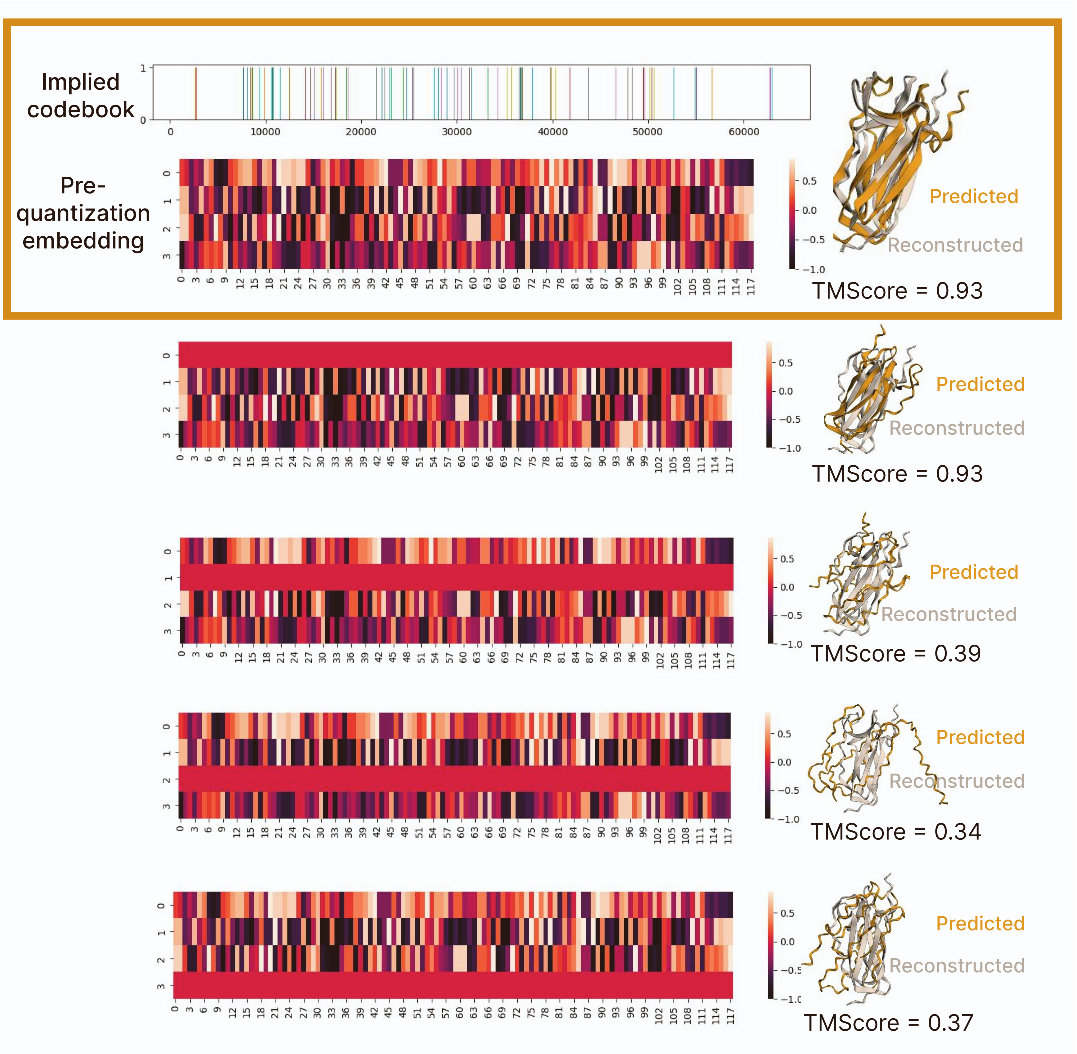

Figure S2: **Understanding token-space representation by corruption.** Exploring effects of token manipulation on the structure space for the $[16, 16, 16, 16]$ FSQ model, by setting some values to 0 just prior to quantization. The yellow box (top) denotes the original pre-quantization embedding and discrete codes representations of all-atom structure.

References

1. Lin, Z., Akin, H., Rao, R., Hie, B., Zhu, Z., Lu, W., Smetanin, N., Verkuil, R., Kabeli, O., Shmueli, Y. et al. (2023). Evolutionary-scale prediction of atomic-level protein structure with a language model. Science *379*, 1123–1130.

2. Mentzer, F., Minnen, D., Agustsson, E., and Tschannen, M. (2023). Finite scalar quantization: Vq-vae made simple. arXiv preprint arXiv:2309.15505.

3. Xu, M., Zhang, Z., Lu, J., Zhu, Z., Zhang, Y., Chang, M., Liu, R., and Tang, J. (2022). Peer: a comprehensive and multi-task benchmark for protein sequence understanding. Advances in Neural Information Processing Systems *35*, 35156–35173.
