# Supplementary material for: Tokenized and continuous embedding compressions of protein sequence and structure
Source: Patterns (N Y). 2025 Jun 13;6(6):101289. doi: 10.1016/j.patter.2025.101289 (PMC12191763; doi:10.1016/j.patter.2025.101289)
Supplement: Document S2. Article plus supplemental information [file mmc2.pdf]

Patterns

Article

Tokenized and continuous embedding compressions of protein sequence and structure

Graphical abstract

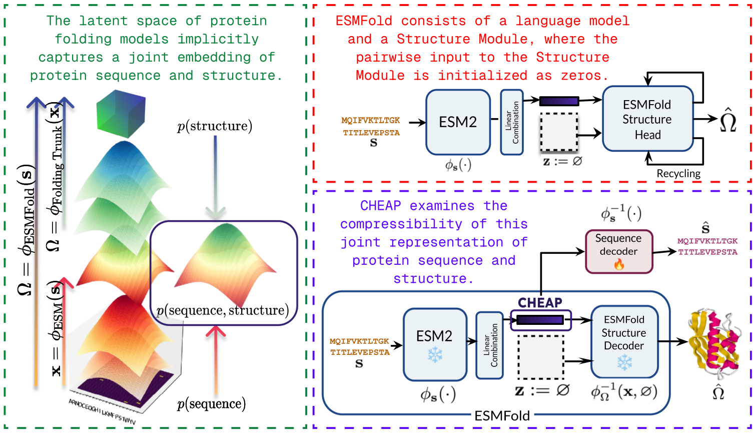

Highlights

- CHEAP is a joint embedding of protein sequence and structure
- ESMFold latent spaces can be compressed by 128× while retaining structural information
- PLMs show massive activations, with some channels far exceeding others in magnitude

Authors

Amy X. Lu, Wilson Yan, Kevin K. Yang, ..., Pieter Abbeel, Richard Bonneau, Nathan C. Frey

Correspondence

amyxlu@berkeley.edu

In brief

This work presents compressed hourglass embedding adaptations of proteins (CHEAP) embeddings, a compact representation that integrates both sequence and structural features of proteins from the ESMFold model. By reducing the channel dimension by up to 128-fold and the embedding length by 8-fold, these embeddings can compactly maintain high-resolution structure and sequence information. CHEAP facilitates diverse applications, including protein design, search, and functional prediction, making advanced protein modeling more accessible. This approach advances the interpretability and utility of large-scale protein representations in computational biology.

Lu et al., 2025, Patterns 6, 101289
June 13, 2025 © 2025 The Authors. Published by Elsevier Inc.
https://doi.org/10.1016/j.patter.2025.101289

Patterns

CellPress
OPEN ACCESS

Article

Tokenized and continuous embedding compressions of protein sequence and structure

Amy X. Lu,[1,2,5,*] Wilson Yan,[1] Kevin K. Yang,[3] Vladimir Gligorijevic,[2] Kyunghyun Cho,[2,4] Pieter Abbeel,[1] Richard Bonneau,[2] and Nathan C. Frey[2]
[1]University of California, Berkeley, Berkeley, CA, USA
[2]Prescient Design, Genentech, South San Francisco, CA, USA
[3]Microsoft Research New England, Cambridge, MA, USA
[4]New York University, New York City, NY, USA
[5]Lead contact
*Correspondence: amyxlu@berkeley.edu
https://doi.org/10.1016/j.patter.2025.101289

THE BIGGER PICTURE Proteins are vital for life, and AI is transforming how we understand and engineer them. Working with protein structural information requires large datasets and powerful computers, and these AI models use advanced algorithms to make accurate predictions. To power these AI tools effectively, we need compact yet meaningful ways to represent proteins. Here, we introduce compressed hourglass embedding adaptations of proteins (CHEAP) embeddings, a method to simplify complex information about proteins so that computers can understand and work with them more easily. CHEAP captures both sequence and structural information from protein-folding models (i.e., ESMFold) into a single compressed representation, a representation of a protein that is more accessible for analysis. This offers a computationally efficient foundation for diverse applications—like designing new proteins, searching databases, and predicting function—thus making complex protein analyses more accessible.

SUMMARY

Existing protein machine learning representations typically model either the sequence or structure distribution, with the other modality implicit. Here, we characterize an embedding of the joint distribution of protein sequence and structure by compressing the latent space of the protein folding model ESMFold. This provides mechanistic interpretability insights, as well as a flexible compressed representation. We term these CHEAP (compressed hourglass embedding adaptations of proteins) embeddings. In continuous compression schemes, the ESMFold latent space can be reduced by factors of 128× along the channel and 8× along the length while retaining structure information at <2 Å scale accuracy and performing competitively on protein function and localization benchmarks. In discrete compression schemes, we construct a tokenized all-atom structure vocabulary that retains high reconstruction accuracy, thus introducing a tokenized representation of an all-atom structure that can be obtained from the sequence alone. CHEAP democratizes representations captured by large models and can enable flexible downstream applications such as generation, search, and prediction.

INTRODUCTION

Structure is an important determinant in scaffolding functions and biomolecular interactions. Real-world manufacturing of proteins requires precise specification of sequence, and most extant functional biomolecules result from evolution in sequence space. Capturing useful information from all available data for proteins may unlock powerful new capabilities for protein design. Protein sequence datasets can be 10^2–10^4 times larger than structural datasets. As of 2024, the PDB[1] structural dataset contains 218,196 samples, while the UniRef50[2] sequence-only dataset contains 63,849,054 samples. Being able to accurately capture high-precision structural information from sequence input is therefore desirable for machine learning applications in biology.

In recent years, sequence-to-structure prediction[3,4] or structure-to-sequence design[5,6] models have become increasingly capable. Such models have been described as "protein structure foundation models,",[7] mirroring a broader paradigm in AI where large models pretrained on large corpora of data can be adapted for an array of downstream tasks.[8] These models capture intricate information that emerges with scale, but in their

 © 2025 The Authors. Published by Elsevier Inc.
This is an open access article under the CC BY license (http://creativecommons.org/licenses/by/4.0/).

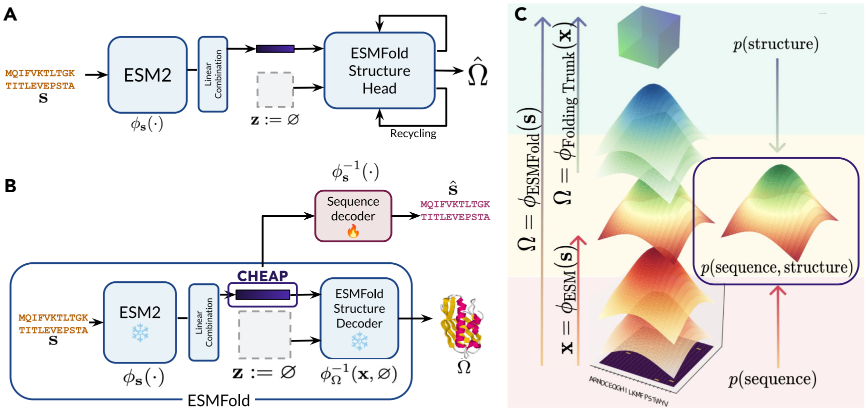

Figure 1. Deriving a joint latent space of structure and sequence using single-sequence sequence-to-structure predictors

(A) Inference time usage of ESMFold, re-interpreted from Lin et al.[4] A protein language model $\phi_s(s)$ and a linear combination of output representations from each layer together map a sequence s of length L to a latent embedding $x \in R^{L \times 1024}$. The structure trunk uses this input to predict an output structure, $\widehat{\Omega} \in \mathbb{R}^3$, along with a $L \times L$ pairwise embedding, z, as input. At inference time, z is simply initialized to zeros and updated in subsequent recycling iterations, whereby outputs of the structure module, s' and z', are reused as inputs.

(B) Harnessing the ESMFold latent space to obtain a joint representation of all-atom structure and sequence from sequence input. To decode the latent as structure (e.g., for latent generation), the original ESMFold structure decoder can be used; to map back to sequence, we separately train a sequence decoder, which achieves 99.7% validation accuracy.

(C) The latent space x can be viewed as a joint embedding of sequence s and structure Ω; since the embedding requires only sequence to derive but can be directly mapped to structure with the frozen ESMFold folding trunk, the embedding encodes both sequence and structural information.

native formulation as prediction models, they are unable to perform generation, similarity search, property prediction, etc., thus limiting their use as a foundation model.

Since sequence-to-structure models map from $p(\text{sequence})$ to $p(\text{structure})$, their intermediate latent space can be viewed as a representation of the joint distribution $p(\text{sequence}, \text{structure})$ (Figure 1B). Of particular interest, ESMFold[4] demonstrates that sequence-to-structure prediction can be built on top of protein language model (pLM) embeddings (Figure 1A). This provides a compact latent space of joint structure and sequence information (Figure 1C).

Naively intercepting this latent space, however, presents numerous challenges. We find that pLM latent spaces contain abnormally high activations in certain channels that persist regardless of the input sequence (Figure 2), rendering them unwieldy for many downstream tasks. Additionally, the intrinsic dimensionality of language models, including pLMs, is often much smaller than the actual channel dimension.[9] Though subtle, embeddings with unnecessarily large dimensions sharply limit their utility. Aside from increasing computational resource demand, they limit the range of possible downstream applications. For example, high-resolution visual synthesis involving larger pixel arrays is generally a more difficult generation task. For protein similarity quantification and search, the dot-product matrix multiplication performed between sequence embeddings has memory constraints that scale in $\mathcal{O}(n^2)$ with the number of channels.[10] Furthermore, existing protein representations typically share the same length L as the original protein[4,11]; however, as transformer[12] architectures are being adopted for a broader range of tasks, it may be computationally desirable to downsample along the length dimension since attention requires $\mathcal{O}(n^2)$ memory with length L. Finally, the compressibility of data provides insight into their complexity and can inform downstream modeling choices; for example, the compressibility of data has been related to the compute-optimal frontier between favoring dataset size as opposed to scaling laws.[13]

In this work, we compress the ESMFold latent space and introduce CHEAP (compressed hourglass embedding adaptations of proteins) representations in two variants: (1) token representations of the discrete biophysical concepts that induce the continuous 3D protein structure and (2) continuous compressed embeddings that investigate the intrinsic dimensionality for tasks of interest. Continuous CHEAP embeddings can retain structural information at Angstrom-scale accuracy and achieve nearly 100% accuracy in retaining sequence information despite reducing the channel dimension by up to 128$\times$ and the length of the embedding (i.e., downsampling) by 8$\times$. Discretized CHEAP tokens also retain high levels of structural information, but unlike concurrent work such as ESM3,[14] CHEAP embeddings can be *obtained from sequence alone*, thus greatly enlarging the size of usable training data for applications requiring structural information. Furthermore, our investigation sheds light on the mechanistic interpretability of increasingly popular pLMs, and on the information content asymmetry between structure and sequence, by demonstrating that structural information is much harder to compress than sequence information in protein embeddings. Code is publicly available at https://github.com/amyxlu/cheap-proteins,[15] and weights are available at https://huggingface.co/amyxlu/cheap-proteins.[16]

RESULTS

Defining a joint structure-sequence latent space

A protein is comprised of a combination of 20 amino acids. Given a protein of length L with one-hot-encoded sequence $\mathrm{s} \in \mathbb{I}^{L \times 20}$ and 3D structure $\Omega \in \mathbb{R}^3$, our goal is to find an embedding that encapsulates the joint distribution $\mathrm{x} \sim p(\mathrm{s}, \Omega)$; that is, the embedding x should both encode sequence information $\mathrm{x} = \phi_s(\mathrm{s})$ and structure information $\mathrm{x} = \phi_\Omega(\Omega)$. We first decompose the mapping as

$$\mathrm{x} = \phi(\mathrm{s}, \Omega) = \phi_s(\mathrm{s}) \circ \phi_\Omega(\Omega).$$

Though we can train separate encoders for $\phi_s(\cdot)$ and $\phi_\Omega(\cdot)$, it is advantageous to leverage large pretrained models to improve performance and reduce computational overhead. In particular, ESMFold demonstrates that structure prediction is possible from pLM representations learned by sequence-masked language

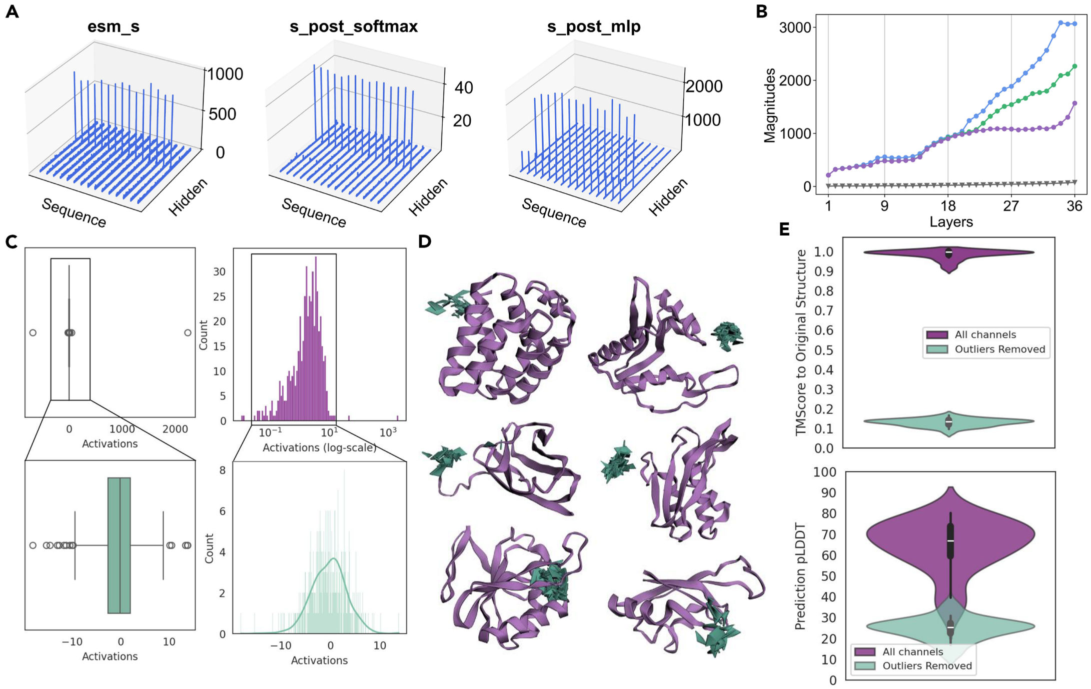

Figure 2. ESM2 contains massive activations in its embeddings

(A) For certain activations, massive activations occur at certain channels regardless of the sequence positions, at channel positions [274, 641, 37]. The pattern persists across layers (Data S1).

(B) Layerwise analysis demonstrating an increase in the magnitude of activations in the 3 billion parameter ESM2 model. Gray denotes the median activation magnitude; blue, green, and purple denote the top 3 largest activation magnitudes per layer output and are significantly larger than the median activation.

(C) Histogram of per-channel means. The total range of embedding values is greatly enlarged by the presence of massive activations. By removing three outlier channels with mean absolute values >20, the resulting latent space is closer to the Gaussian assumption necessary for diffusion.

(D) Structure prediction results after setting the three outlier channels to zero. Purple denotes the original ESMFold prediction, and green denotes the ESMFold prediction after setting outlier channels to zero. Removing 3 channels with massive activations causes performance to deteriorate.

(E) Quantitative assessment of the effect of outlier channel removal on structure prediction, measured by the TM-score (measure of structure prediction accuracy) and pLDDT (measure of model confidence), visualized for a random sample of 64 CATH structures. Model performance deteriorates by both metrics.

modeling, which reduces computation requirements and improves performance for orphan proteins[4]; we use this model as a representative for sequence-to-structure models. The ESM2-3B language model $\phi_{\mathrm{ESM}}(\mathrm{s})$ embeds sequence s into a 2,560D sequence representation, which is then mapped via linear projections to an embedding $\mathrm{x} \in \mathbb{R}^{L\times 1024}$. This is used as input to the folding trunk $\phi_{\Omega}^{-1}(\mathrm{s},\mathrm{z})$, where $\mathrm{z} \in \mathbb{R}^{L\times L\times D_z}$ represents pairwise contacts. An overview of the original ESMFold model with notations used in this work is shown in Figure 1A.

A key observation is that at inference time, it is empirically sufficient to initialize input z as an array of zeros to obtain high-quality structure predictions as reported in Lin et al.,[4] such that $\Omega = \phi_{\Omega}^{-1}(\mathrm{x},\mathrm{z})$ becomes $\widehat{\Omega} = \phi_{\Omega,\mathrm{Inference}}^{-1}(\mathrm{x})$. That is to say, x is a direct inverse mapping from 3D structure Ω to a high-dimensional latent space. This sequence embedding x, therefore, obtained as the latent space of the ESMFold model, can be seen as our embedding of the joint distribution of $\phi(\Omega,\mathrm{s})$:

$$\mathrm{x} = \phi_{\mathrm{s}}(\mathrm{s}) \quad \text{linear combination of ESM2 representations} \qquad \text{(Equation 1)}$$

$$\Omega = \phi_{\Omega}^{-1}(\mathrm{x}, \varnothing) \quad \text{ESMFold structure module.} \qquad \text{(Equation 2)}$$

Note that x is not precisely the same as ESM2 embeddings since in ESMFold, ESM2 representations from each layer are linearly combined with trainable weights to create the x embedding that is input to folding trunk ϕ_{Ω}^{-1}. See Data S1 for a code sketch to obtain $\mathrm{x} \sim p(\mathrm{s},\Omega)$, and a graphical description is shown in Figure 1B.

We would also like to map this latent space back to sequence and structure space for assessing compression performance and for generative modeling. To decode this latent space back to protein sequences, a sequence encoder $\mathrm{s} = \phi_{\mathrm{s}}^{-1}(\mathrm{s})$ is trained separately. We achieve a per-token accuracy of 99.7% on a randomly partitioned holdout set (detailed in training), most likely because the task of mapping a language model embedding to 21 classes is fairly simple and closely related to how the ESM2 model was trained. To obtain structure from embedding, we can simply use the frozen ESMFold folding trunk, $\Omega = \phi_{\Omega}^{-1}(\mathrm{x})$. If generative model parameters θ are learned to approximate $p_\theta(\mathrm{x})$, then at inference time, after sampling $\tilde{\mathrm{x}} \sim p_\theta(\mathrm{x}) = p_\theta(\mathrm{s},\Omega)$, we can generate new $\tilde{\mathrm{s}} = \phi_{\mathrm{s}}^{-1}(\tilde{\mathrm{x}})$ and $\tilde{\Omega} = \phi_{\Omega}^{-1}(\tilde{\mathrm{x}})$ using the decoders $\phi_{\Omega}^{-1}(\mathrm{x})$ and $\phi_{\mathrm{s}}^{-1}(\tilde{\mathrm{x}})$ defined above.

ESMFold latent space contains massive activations

We find that the latent space of ESMFold is pathologically disorganized, whereby some channels have mean values up to

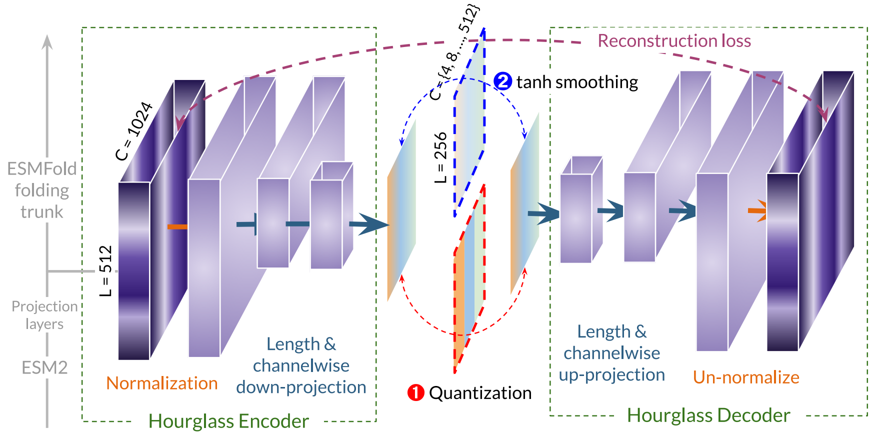

Figure 3. The hourglass protein compression transformer model

For a protein with 512 amino acids, the embedding right before the ESMFold structure encoder has dimensions $512 \times 1,024$. To correct for massive activations, the embedding is normalized using the statistics of each channel (per-channel normalization). The encoder condenses the channel dimensions with a linear projection and a linear downsampling operation along the length dimension. In the bottleneck layer, we examine methods for obtaining both discrete and continuous compressed embeddings.

$3,000\times$ larger than others. This is consistent with reports of this phenomenon in large transformer[12] models for text and image modalities, where they are sometimes referred to as massive activations[17] or outlier features.[18] Figure 2A visualizes embedding values at various intermediate layers between the ESM2 output and the folding trunk, following variable notations in Data S1.

Massive activation accumulation begins in the ESM2 language model. The top values in any given layer can be up to $3,000\times$ larger than the median activation value in a given layer (Figure 2B). Figure 2C visualizes the means of each channel. When three outlier channels with massive activations are removed, the resulting distribution is more organized; however, removing these three features alone causes structure prediction performance to deteriorate, as seen qualitatively (Figure 2D) and quantitatively (Figure 2E), bringing the template modeling score (TM-score) of predictions from an average of 0.97 down to 0.14.

Massive activations limit the flexibility with which large protein models can be used as foundation models. Dettmers et al.[18] examine 8-bit model quantization to reduce memory usage during inference, which is rendered difficult due to the expanded numerical range caused by the 0.01% of abnormally high values. Massive activations have also been observed to dominate attention patterns and have been suggested to act as an implicit bias term.[17] Given that attention mechanisms have been shown to learn protein contacts,[19] these massive activations may play interpretable roles in how structure information emerges from pLMs; we leave the specifics of this investigation as future work. We combat this by applying a per-channel normalization scheme, which is described in per-channel normalization.

A neural compression architecture for protein embeddings

The equivalence between data compression and data distribution representation learning is foundational to machine learning.[20] Biological data are comprised of both signal and noise, not all of which is necessary to understand their structure or function; despite this, data compression remains understudied for proteins. Our work aims to bridge this gap by studying protein embeddings as an instance of neural compression. We design the hourglass protein compression transformer (HPCT) architecture, an autoencoder with a bottleneck layer, further detailed in hourglass compression transformer and visualized in Figure 3, for protein embedding compression.

Proteins have different lengths, rendering the ResNet[21] autoencoder architecture used in vision a poor choice. Inspired by Nawrot et al.,[22] HPCT (Algorithm 1) includes a linear downsampling operation (Algorithm 2), which "shortens" the embedding $x \in \mathbb{R}^{L \times D}$ to $x \in \mathbb{R}^{\frac{L}{S} \times D}$ using a shortening factor of S. A linear projection further compresses the channel dimension. The model is trained end to end with a mean-squared error (MSE) reconstruction loss, such that it is trained to maximally preserve the original content across the bottleneck layer. At inference time, the output of the bottleneck layer is used as the compressed embedding. In early experiments, we also applied a sequence-space loss (i.e., cross-entropy loss constraint that the decoded amino acid corresponds to the original) and structure-space loss (backbone frame-aligned point error[3]) but did not find these to substantially improve performance.

Continuous versus discrete compressions

Performing data compression also has practical motivations. For example, in high-resolution image synthesis, the increased number of pixels requires more FLOPS (floating point operations per second), which increases compute costs. High-resolution generation also generally has lower performance compared to lower resolution generation. To combat this, latent diffusion models (LDMs)[23] perform diffusion modeling[24,25] in a continuous compressed latent space learned by a vector-quantized generative adversarial network (VQ-GAN)[26] encoder. Another successful technique is encoding continuous images as discrete tokens and generating tokens using an autoregressive model or by sampling from mask tokens.[27–29] We aim to make contact with these paradigms by investigating how to build the necessary autoencoder for protein data.

All experiments use the embedding x defined in defining a joint structure-sequence latent space, though the architecture can be used for any sort of embedding compression. The dimension of x defined in defining a joint structure-sequence latent space is $L \times 1,024$ for a sequence with length L. Note that the embedding dimension has already been reduced from the $L \times 2,560$ ESM-3B embeddings. For a protein sequence of length 512, the size of this embedding maps to high-resolution image synthesis. As a point of comparison, LDMs[23] compress input images of size $256 \times 256 \times 3$ down to $64 \times 64 \times 4$ before diffusing.

CellPress
OPEN ACCESS

Algorithm 1. Hourglass compression transformer

Require: embedding $a \in \mathbb{R}^{L \times D}$, mask $m^{\text{Length}[L]} := \{1,0\}^L$, shortening factor $S \in \mathbb{Z}$, downprojection factor $K \in \mathbb{Z}$, downprojection $W_d \in \mathbb{R}^{D \times \frac{D}{K}}$, upprojection $W_u \in \mathbb{R}^{\frac{D}{K} \times D}$
procedure HourglassCompression(a, m)
  $a \leftarrow$ Transformer(a, m)
  $a' \leftarrow$ LinearDownsampling(a, S) ▷ *Alg. 2*
  $m' \leftarrow \sum_S m^{\text{Length}[L] \rightarrow [\frac{L}{S}]} > 0$ ▷ *Reduce*
  $a' \leftarrow$ AttentionResampling(a', a, m')
  $c \leftarrow W_d a'$
  if quantize **then**
    $c \leftarrow$ Bottleneck(c, m')
  else
    $c \leftarrow$ Tanh(c)
  end if
  $a' \leftarrow W_u c$
  $a \leftarrow$ LinearUpsampling(c', m')
  $m \leftarrow m'^{\text{Length}[\frac{L}{S}] \rightarrow [L]} > 0$ ▷ *Repeat*
  $a \leftarrow$ AttentionResampling(a, a', m)
  $a_{\text{reconstructed}} \leftarrow$ Transformer(a, m)
return $a_{\text{reconstruction}} \in \mathbb{R}^{L \times D}$, $c \in \mathbb{R}^{\frac{L}{S} \times \frac{D}{K}}$
end procedure

At the bottleneck layer of HPCT, we prefer either a quantization step at the bottleneck to obtain *discrete* compressed embeddings or a tanh step to obtain *continuous* compressed embeddings. The tanh layer constricts embedding values to between $[-1, 1]$ and improves its utility (e.g., for latent diffusion); in early experiments, we found that it does not degrade performance.

Compression evaluation metrics

In the following sections, we assess continuous and discrete compression performance. To assess in-distribution structure reconstruction, we train and assess on the CATH[30] database, though a sequence-only dataset such as Pfam[31] or UniRef[2] can also be used. Mirroring the usage of perceptual losses for assessing compression in images,[26] which favors retention of visual details necessary for downstream tasks as opposed to pure reconstruction, we examine reconstruction performance in the structure and sequence space in addition to the MSE between input and reconstruction. The TM-score is a backbone-only metric of structure reconstruction, while the root-mean-squared distance (RMSD) is a more fine-grained measure between atom positions. The root-mean-squared paired distance (RMSPD) is similar to RMSD but uses pairwise distances rather than atom positions; we use it to assess superimposition-free reconstruction accuracy. Sequence reconstruction accuracy is the fraction of token matches after decoding back to sequence space, divided by the number of tokens in the sequence. As reference points for interpreting structure reconstruction results, we note that 1.34 Å is the inter-carbon distance taken from Jumper et al.,[3] and the range 0.8–4.1 Å is the range of experimental resolutions for the original structures.

A discrete vocabulary of protein structure and function

Quantization or tokenization here refers to learning an encoder that maps an input to a discrete representation. Given a d-dimensional input $a \in \mathbb{R}^d$ and a pre-specified codebook size C, the goal is to find a set of integers from $\mathcal{C} \in \{0, 1, \ldots, C\}$ that represents the input. Intuitively, though pixel inputs of the visual world are continuous, many discrete concepts exist, such as color, shape, and size; similarly, though structures exist in the continuous 3D space, the biophysical concepts that cause the shape might manifest discretely. Furthermore, obtaining a discrete vocabulary of representations allows scaling infrastructure for large language models to be applied.

Tokenized representations of protein structure are an area of limited investigation. Foldseek[32] introduces the 3Di vocabulary for its search algorithm, though this is limited to 20 amino acids, and it is known that codebook size (i.e., the number of discrete options) is a crucial factor in representation quality. Some concurrent works have also examined characterizing a tokenization scheme for structure.[33–35] However, these methods are learned

Algorithm 2. Hourglass encoder shortening by linear downsampling

Require:
  $a \in \mathbb{R}^{L \times D}$,
  integer shortening factor S,
  $W \leftarrow W \in \mathbb{R}^{(SD) \times D}$,
  procedure LinearDownsampling(a, S)
    $a \leftarrow a \in \mathbb{R}^{\frac{L}{S} \times (SD)}$ ▷ Reshape
    $a \leftarrow aW$ return $a \in \mathbb{R}^{\frac{L}{S} \times D}$
end procedure

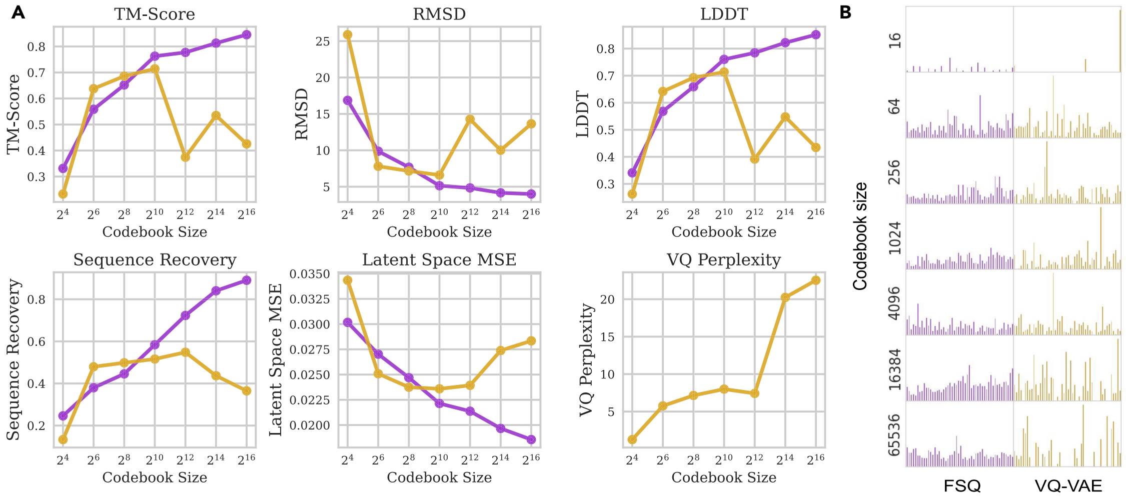

Figure 4. Assessing structure, sequence, and latent reconstruction across codebook sizes for the FSQ and VQ-VAE methods

(A) Across all metrics, FSQ outperforms VQ-VAE at codebook sizes greater than 2^{10}. Blue arrows denote metrics where lower is better, and red arrows denote metrics where higher is better. VQ-perplexity refers to the codebook selection prediction in VQ-VAE, which does not apply to FSQ.

(B) Examining codebook utilization for FSQ and VQ-VAE. We find that codebook utilization is generally more favorable using FSQ (left), though we do not find catastrophic codebook collapse for VQ-VAE above a codebook size of 2^4.

on structural input, unlike the scheme here, which can be used on sequence-only input. ESM3[14] constructs separate discrete vocabularies for structure, function, and sequence, as opposed to directly crafting a single discrete embedding that can be decoded into multiple modalities.

While VQ variational autoencoders (VQ-VAEs)[36] are a popular choice, they can be difficult to optimize and are prone to "codebook collapse," whereby a few codes are over-utilized and require specific interventions.[37–40] Thus, we also investigate the recently proposed FSQ (finite scalar quantization)[41] approach, which obtains token representations without quantizer parameters. Details of the VQ-VAE and FSQ methods are described in quantization methods.

The choice of codebook size relates to the bits of information content in the data and the degree to which information loss is tolerable for the downstream task. It is unclear how to best select this for an all-atom protein structure and sequence. We find that while VQ-VAE outperforms FSQ on codebook sizes smaller than $2^8 = 256$, FSQ consistently outperforms VQ-VAE on latent space reconstruction MSE, structure space metrics (both the backbone-only TM-score and the all-atom RMSD), and sequence space metrics (Figure 4). This is consistent with image experiments in Mentzer et al.[41] FSQ generally has more evenly distributed codebook usage, especially for larger codebooks. This is reflected in the high VQ-perplexity (perplexity when selecting a code during the VQ-VAE quantization process) as codebook size C increases, a metric that does not apply to FSQ.

Continuous compression performance on structure reconstruction and function prediction

Our experiments empirically validate the hypothesis that many channel dimensions in protein representations are extraneous and that the intrinsic dimensionality is most likely lower. Figure 5A examines the ability of compressed embeddings to retain structural information, examined both with and without length downsampling. Even with a 32× compression factor down to only 32 channels, the model is still able to achieve <1.34 Å RMSD in structure fidelity compared to the original prediction. When shortening by a factor of two, one can attain a <1.34 Å RMSD at 64 channels or higher. For sequences, there is nearly no drop in reconstruction performance until compressing to less than 8 channels. Interestingly, the largest drops in performance are found after compressing to 4 channels by both structure and sequence reconstruction accuracy, which may be related to the average number of atoms in amino acid residues. The pairwise RMSD, which is independent of superimposition, is generally lower than the RMSD, suggesting that some atomic distances may be due to a loss of precision in trivial 3D superimposition rather than insufficiently learning protein information. A visual depiction of how compression changes the predicted structure is shown in Figure 5C. Figure S1 shows the t-distributed stochastic neighbor (t-SNE) results for embeddings at different levels of compression. Compression results on sequence-only datasets (Pfam and CATH sequences) are shown in Table S2.

We further examine function and localization prediction using datasets from the PEER (protein sequence understanding) benchmark[42] for embeddings at different compression levels, described in Figure 6. Function prediction performances seem to suffer more with compression than structure and sequence recovery. Training describes the details of the training procedure. This is consistent with recent works demonstrating that pLMs fail in downstream tasks that do not involve structure.[43] For some tasks, such as β-lactamase activity prediction and subcellular localization, compression seems to be more impactful than tasks such as solubility. We show comparisons against other methods along with additional benchmarks in Tables S3 and S4. In structure-related tasks, compressed embeddings can compare competitively with other methods, sometimes exceeding their performance.

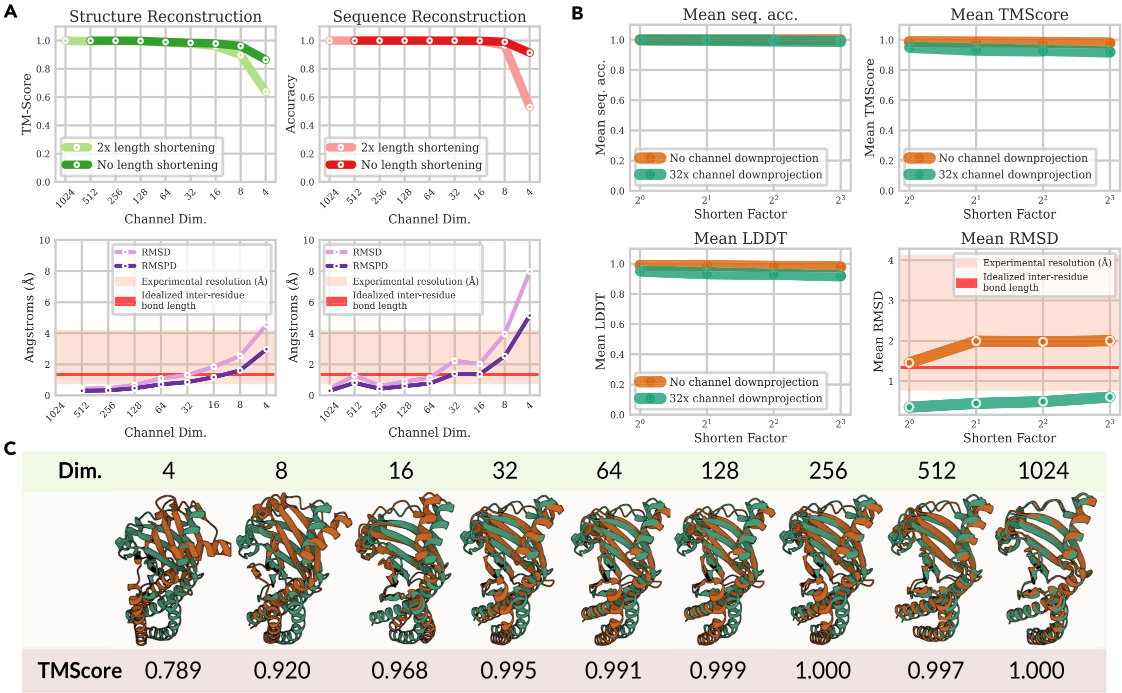

Figure 5. Assessing compressibility of structural and sequence information in protein embeddings

(A) Examining compressibility of backbone structure (TM-score), sequence identity (sequence recovery accuracy), all-atom structure (RMSD), and superimposition-free all-atom structure (pairwise RMSD). Sequence information and backbone information can be preserved nearly perfectly even after reducing down to only 8 channels; all-atom structure RMSD similarly achieves <1.34 Å accuracy at 16 channels or less.

(B) Length information can be compressed while preserving sequence and backbone information even after reducing channel dimensions to 32.

(C) Visual overlay of structural information compressibility. Even with only 4 embedding channels, secondary structures can be preserved after reconstruction.

Interpolation and noising in the latent space

To examine the smoothness of the latent space, we perform linear interpolations in the embedding space and examine how this manifests in the structure and sequence space. For two real proteins in the CATH dataset, we obtain the CHEAP embedding representation and perform a linear interpolation as $x' = tx_1 + (1 - t)x_2$. We then transform this embedding back to the sequence and structure space and examine their similarities to the proteins that they were interpolating between and measures of the "naturalness" of the produced sequence via perplexity and the structure via predicted local distance difference test (pLDDT) (naturalness evaluation).

Figure 7A demonstrates reconstruction results in the latent space. We find that the unprocessed ESMFold latent space is not very "smooth"—that is, despite changes linearly in continuous space, the discrete changes in the sequence space are limited, as visualized by the sequence accuracy to the end sequence. A similar case is observed for structures. After the per-channel normalization (per-channel normalization) and compressing down to 8 channels, the sequence space is

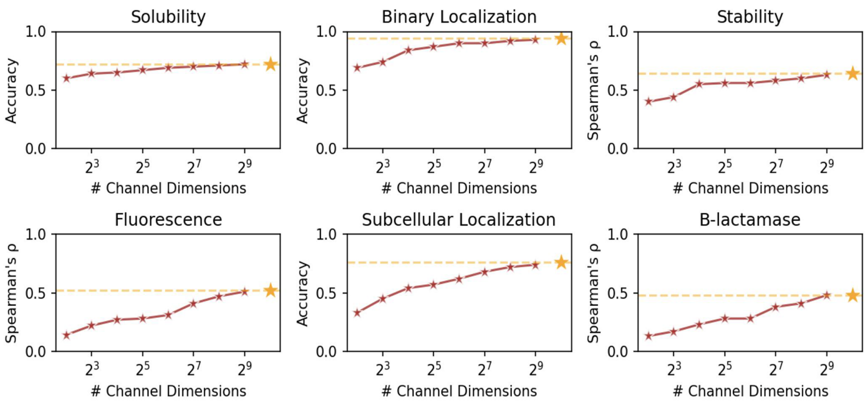

Figure 6. Using linear probes to evaluate compressibility of functional information

The title of each graph refers to the task evaluated. For all results, a 2× lengthwise shortening was applied. Orange star denotes uncompressed performance. Unlike structure and sequence, performance degrades more gradually for some functions (e.g., β-lactamase activity), though the performance drop is less pronounced for other tasks (e.g., solubility).

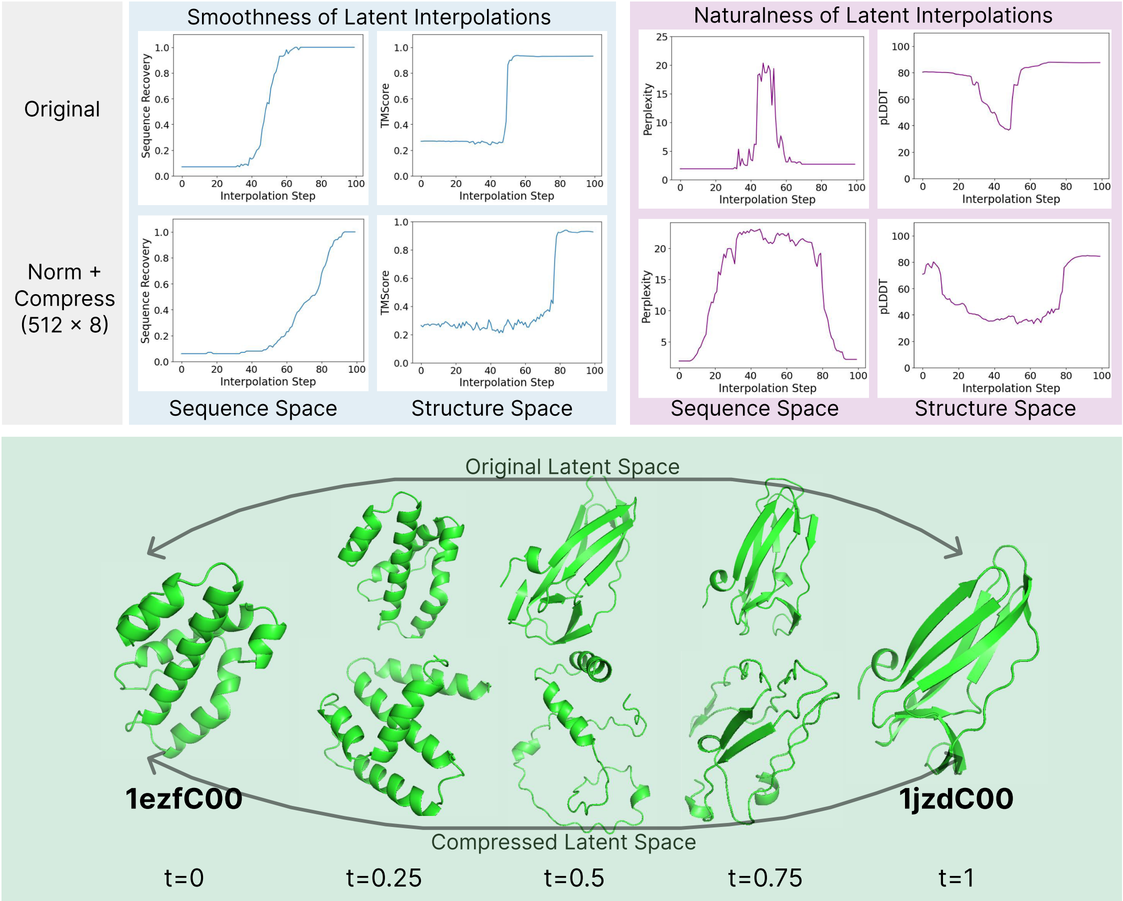

Figure 7. Comparing linear interpolation in various embedding spaces as empirical assessment of latent space geometry

(Top) To examine how smoothly sequence changes as a result of interpolating in a given latent space, we decode interpolations back to sequence/structure and examine sequence recovery accuracy (↓) and backbone TM-score (↑) to the final sample. Despite moving linearly in embedding space, the actual sequence and structure samples that embedding interpolations map to remain tightly coupled to proteins seen in the training set. Interpolations typically occupy regions of low perplexity (↓) and high structure prediction confidence (pLDDT, ↑). Our compression and normalization schemes help improve the smoothness of interpolations and raggedness of the approximated fitness landscape. (Bottom) Visual examination of structure reconstructions of linear embedding interpolations.

more smooth and changes more gradually with linear interpolation in the embedding space; however, the structure space remains less smoothed out. Despite being a smoother interpolation between the two natural proteins, these proteins are less "natural," though it may better reflect the ragged nature of the hypothesized protein fitness landscape.[44] This may reflect the classic quality-versus-diversity trade-off but also raises questions on the geometry of the protein structure fitness landscape and whether a more rugged or sharp landscape is more suitable for exploration. Figure 7C provides a visual of how the structures change when interpolating in the original latent space versus the compressed latent space. The jumps are more gradual when interpolating in the compressed latent space and then transforming back to the embedding with massive activations; however, they also tend to be more unstructured.

For the tokenized latent space, we perform a mirror of this experiment by corrupting some latent tokens to a random token and examining how this manifests in the structure space (Figure S2). As expected, altering the tokens also results in disruptions in the structure space. However, not all alternations result in the same degree of disruption.

DISCUSSION

In this work, we characterize how the latent space of large protein sequence-to-structure models can represent the joint distribution of sequence and structure. We further identify pathologies of massive activations in ESMFold; indeed, the original paper uses a linear combination of representations from all layer outputs of the language model rather than the final embedding, and though no explicit justification or ablations were given in Lin et al.,[4] our findings of how pathological massive activations accrue across layers may contribute to why representations from intermediate layers might be as or more informative than the final layer embedding. We also find that protein sequence embeddings are highly compressible and develop the transformer-based HPCT autoencoder architecture. Sequence information can be captured by embeddings of only 8 channels. All-atom structural information is less compressible than sequence

information, but nonetheless, remarkable reconstruction performance can be obtained by embeddings that undergo 128× channelwise compression and 8× lengthwise compression between amino acids. We also develop a structure tokenization dictionary, which can be obtained from sequence alone, unlike concurrently proposed structural tokenizers.[33–35] Further, we show that FSQ is more effective than the dominant VQ-VAE approach (which has also been used for proteins by recent works[14]), especially for large codebooks.

Building a representation of the joint distribution of structure and sequence has many desirable traits for generation, search, and representation learning. The CHEAP series of embeddings is a compact joint embedding of the latent space that can be flexibly employed. Furthermore, our empirical results raise interesting theoretical questions on the information content and geometry of protein embeddings. The compressibility of embeddings with respect to sequence identity and structural precision corroborates empirical investigations that the intrinsic dimensionality of existing structure foundation models is probably much lower,[9] and there may be over-parameterization in existing models. The lack of a clear pattern for linear probes relating to function further illustrates the poorly understood nature of how protein embeddings capture relevant information.

An alternative means of achieving this is to train and/or fine-tune ESMFold (or another sequence-to-structure model) end to end with the bottleneck layer and with a channel normalization before it. We hypothesize that this should improve the properties desirable for empirical downstream use without compromising structure prediction quality, though due to computational resource limitations, we leave this as future work.

METHODS

Per-channel normalization

To address the issue of massive activations, as shown in Figure 2, we use a per-channel normalization scheme. Conventionally, following Ho et al.,[24] image generation works rescale image pixel values from $[0,256]$ to $[-1,1]$. Subsequent works find this bounding strategy to be important to performance, such as for color saturation.[45] To remedy channel-specific massive activations, the embeddings are processed as

$$\mathrm{x}' = \frac{\mathrm{x} - \mathrm{x}_{\min}}{\mathrm{x}_{\max} - \mathrm{x}_{\min}} \times ((c_{\max} - c_{\min}) + c_{\min}),$$

where $\mathrm{x}_{\min}$ and $\mathrm{x}_{\max}$ are vectors with shape $(1,024)$ and broadcasted along length dimension. They denote statistics calculated independently for each channel. This prevents outlier channel values from dominating the normalization. In experiments, we choose $c_{\min} = -1$ and $c_{\max} = 1$.

Visual inspection shows that after this transformation, the distribution better befits the Gaussian distribution needed for diffusion, as visualized in Figure 2. A random subset of 5,000 samples from CATH[30] is used, but we find that these mean and standard deviation statistics vary little across datasets such as CATH, UniRef,[2] and Pfam,[31] consistent with the general observation that massive activations are not input specific.[17] In simple diffusion experiments, this greatly improves the stability of training.

Hourglass compression transformer

A sketch of the hourglass compression transformer is as follows, inspired largely by Nawrot et al.[22] Linear downsampling layers serve to shorten the embedding lengthwise, while the downprojection reduces the channel dimension. The attention resampling is an attention layer that attends to both the pre- and post-shortening embeddings. This is similar to the approach in LDMs, where compressed latent space is taken to be the continuous representation just prior to the VQ layer (except we replace the VQ with FSQ in this case).

Though different operations may be used for $g(x)$, we find a simple linear downsampling to work well (Algorithm 2), which can be seen as a convolution with a filter size and stride both equal to S. Since the original model is designed for sequence-to-sequence tasks rather than compression, we remove the skip connections that would make the solving the reconstruction task trivial. Additionally, we add a projection layer along the channel dimension after each shortening operation. At training time, $x_{\mathrm{reconstructed}}$ is used for calculating the MSE reconstruction loss, and at inference time, the output of the encoder is used as the compressed representation, with additional processing in the bottleneck, depending on if the compression is discrete or continuous. Linear downsampling consists of a flattening operation, followed by a learned downsampling weight matrix, and finally reshaping back to L/S, where L is the original length and S is the shortening factor.

Naturalness evaluation

For evaluating sequence naturalness with perplexity, we use the autoregressive RITA XL model.[46] Intuitively, perplexity can be seen as a measure of how "confused" a model is when selecting the next token. If a sequence is in distribution with the natural proteins that the model was trained on, its perplexity will be lower since the model can make sense of the patterns. Conversely, if a sequence out of distribution (e.g., is not a valid protein) and learned patterns from natural proteins cannot help the model discern what the next token should be, then the perplexity increases.

For evaluating structure naturalness, we use the pLDDT score, which is returned by the ESMFold prediction head along with the all-atom structure. The metric was introduced in AlphaFold2[3] and is a predicted measurement of the model's certainty in the structure at that particular position. Similar to the reasoning behind perplexity as a metric of naturalness, a high pLDDT suggests that a metric is in distribution with the natural proteins that were seen during sequence-to-structure training. Note that pLDDT tends to favor structured regions and is typically low for disordered regions, even in real proteins.

Quantization methods

VQ-VAE

The VQ-VAE[36] learns a discrete, compressed semantic representation of the input, typically of images. In the forward pass, the encoder h_e produces a continuous feature representation of input x. Then, each feature vector is mapped to a discrete code in the codebook space, $\mathcal{C}$, where each discrete code is associated with a continous vector e_i. The assembled array of learned codes, their vector embeddings $z = \{e_1, e_2, \ldots e_{|\mathcal{C}|}\}$, and their corresponding features are fed into the decoder $h_q(z)$.

Since the quantization operation is not differentiable, the straight-through estimator (STE)[47] is used by copying the gradients from the decoder input to the encoder output. The codebook is selected via a nearest-neighbor search in Euclidean space; auxiliary losses are introduced to pull the code vectors toward the unquantized encoder outputs. As in autoencoder training, a reconstruction loss between output and input is also used. The complete VQ-VAE loss is

$$L_{VQ} = \log p(x|h_q(z)) + ||\text{sg}[h_e(x)] - z||_2^2 + \beta||h_e(x) - \text{sg}[z]||_2^2.$$

Finite scalar quantization

Rather than using a nearest-neighbor search to choose a code, FSQ directly quantizes the continuous encoder representations $z \in \mathbb{R}^d$ into L bins:

$$z = h_e(x), z \in \mathbb{R}^d \quad \text{continuous encoder representation,} \quad \text{(Equation 3)}$$

$$\hat{z} = \tanh(z) \quad \text{bound to } [-1,1], \quad \text{(Equation 4)}$$

$$\hat{z} = \text{round}(\lfloor (L/2) \rfloor \cdot \hat{z}) \quad \text{discretize to } L \text{ bins and round to nearest integer.} \quad \text{(Equation 5)}$$

The predetermined bin L is selected to be small relative to VQ-VAE codebook sizes. The implicit codebook size $|\mathcal{C}|$, however, comes from the combinatorial possibilities arising from using one of L integers at each of the D channels. For z with d channels, there are d associated integer representations, and thus $|\mathcal{C}| = L^d$. A large implicit codebook can thus be achieved while forcing all codes to be used. The specific FSQ levels used are shown in Table S1.

Training

Compression model training

For all continuous compression models, we use a learning rate of 8^{-5} with the AdamW optimizer and 5,000 warmup steps. For discrete compression models, we use a learning rate of 1^{-4}, with the AdamW optimizer and 1,000 warmup steps. Learning rate schedules are selected based on hyperparameter tuning on a holdout validation set. Train and test sets were split randomly. CATH was used for all experiments, with the exception of the experiments in Table S2.

Sequence decoder

For the sequence decoder, we use a fully connected network with two layers and a hidden size of 1,024. This network is able to converge relatively fast. Though ESM2 was already trained with a masked token objective and thus has pretrained weights that map the language model output to logit space, we opt for retraining since our defined embedding is intercepted after the linear projection layers (with 1,024 dimensions), whereas the original ESM2-3B model has 2,580 dimensions. The output has 21 classes (20 amino acids in addition to the unknown residue placeholder). In early experiments, we also tried using a transformer architecture for this decoder but found that the over-parameterization actually decreases performance.

Function prediction

For results in Figure 5C and Table S3, we use the dataset splits and tasks from Xu et al.[42] For all compressed embeddings, we add a trainable projection layer back to 1,024, such that the size of the downstream fully connected network used for prediction is the same size for all compared networks. A mean pool is applied to the embeddings, with a 2-layer multilayer perceptron used as the downstream probe.

RESOURCE AVAILABILITY

Lead contact

All requests for addition information should be directed to the lead contact, Amy X. Lu (UC Berkeley, Prescient Design) (amyxlu@berkeley.edu).

Materials availability

No reagents were used in this study.

Data and code availability

Our source code is available on GitHub at the GitHub repository and is deposited at Zenodo at https://doi.org/10.5281/zenodo.15128472. Model weights are available on HuggingFace at https://doi.org/10.57967/hf/5067.

ACKNOWLEDGMENTS

The authors thank Mingjie Sun for productive discussions on massive activations in large language models and help producing Figure 2A. The authors additionally thank Sidney Lisanza, Daniel Severo, Igor Mordatch, Andrew Leaver-Fay, and Lian Huang for discussion and comments. A.X.L. is supported in part by the NSERC PGS-D scholarship and Nissan.

AUTHOR CONTRIBUTIONS

A.X.L. and K.K.Y. initiated investigations of ESMFold intermediate layer activations. A.X.L. and W.Y. designed compression model architectures and training scheme. A.X.L. and N.C.F. designed evaluations and analyzed results. A.X.L. wrote code and ran experiments for model training and evaluations. All authors provided guidance throughout. A.X.L. wrote the initial manuscript draft. All authors revised the final manuscript.

DECLARATION OF INTERESTS

A.X.L., V.G., K.C., R.B., and N.C.F. are or were employees of Genentech, Inc., a member of the Roche Group. W.Y. is an employee of Google LLC, a subsidiary of Alphabet, Inc. K.K.Y. is an employee of the Microsoft Corporation. P.A. holds concurrent appointments as a professor at UC Berkeley and an Amazon Scholar; this paper describes work performed at UC Berkeley and Genentech and is not associated with Amazon.

SUPPLEMENTAL INFORMATION

Supplemental information can be found online at https://doi.org/10.1016/j.patter.2025.101289.

Received: November 17, 2024
Revised: February 5, 2025
Accepted: May 15, 2025

REFERENCES

1. Berman, H.M., Westbrook, J., Feng, Z., Gilliland, G., Bhat, T.N., Weissig, H., Shindyalov, I.N., and Bourne, P.E. (2000). The protein data bank. Nucleic Acids Res. *28*, 235–242.
2. Suzek, B.E., Wang, Y., Huang, H., McGarvey, P.B., and Wu, C.H.; UniProt Consortium (2015). UniRef clusters: a comprehensive and scalable

alternative for improving sequence similarity searches. Bioinformatics *31*, 926–932.

3. Jumper, J., Evans, R., Pritzel, A., Green, T., Figurnov, M., Ronneberger, O., Tunyasuvunakool, K., Bates, R., Žídek, A., Potapenko, A., et al. (2021). Highly accurate protein structure prediction with AlphaFold. Nature *596*, 583–589.
4. Lin, Z., Akin, H., Rao, R., Hie, B., Zhu, Z., Lu, W., Smetanin, N., Verkuil, R., Kabeli, O., Shmueli, Y., et al. (2023). Evolutionary-scale prediction of atomic-level protein structure with a language model. Science *379*, 1123–1130.
5. Dauparas, J., Anishchenko, I., Bennett, N., Bai, H., Ragotte, R.J., Milles, L. F., Wicky, B.I.M., Courbet, A., de Haas, R.J., Bethel, N., et al. (2022). Robust deep learning–based protein sequence design using ProteinMPNN. Science *378*, 49–56.
6. Hsu, C., Verkuil, R., Liu, J., Lin, Z., Hie, B., Sercu, T., Lerer, A., and Rives, A. (2022). Learning inverse folding from millions of predicted structures. In Proceedings of the 39th International Conference on Machine Learning, *162*, pp. 8946–8970.
7. Wang, J., Watson, J.L., and Lisanza, S.L. (2024). Protein design using structure-prediction networks: Alphafold and rosettafold as protein structure foundation models. Cold Spring Harbor Perspect. Biol. *16*, a041472.
8. Bommasani, R., Hudson, D.A., Adeli, E., Altman, R., Arora, S., von Arx, S., Bernstein, M.S., Bohg, J., Bosselut, A., Brunskill, E., et al. (2021). On the opportunities and risks of foundation models. Preprint at arXiv. https://doi.org/10.48550/arXiv.2108.07258.
9. Valeriani, L., Doimo, D., Cuturello, F., Laio, A., Ansuini, A., and Cazzaniga, A. (2024). The geometry of hidden representations of large transformer models. Adv. Neural Inf. Process. Syst. *36*, 51234–51252.
10. Boger, R., Lu, A., Chithrananda, S., Yang, K., Skopintsev, P., Adler, B., Wallace, E., Yoon, P., Abbeel, P., and Doudna, J. (2023). Toph (true retrieval of proteins homologs): Adapting a contrastive question-answering framework for protein search. In ICML workshop on computational biology.
11. Elnaggar, A., Heinzinger, M., Dallago, C., Rehawi, G., Wang, Y., Jones, L., Gibbs, T., Feher, T., Angerer, C., Steinegger, M., et al. (2022). Prottrans: Toward understanding the language of life through self-supervised learning. IEEE Trans. Pattern Anal. Mach. Intell. *44*, 7112–7127.
12. Vaswani, A., Shazeer, N., Parmar, N., Uszkoreit, J., Jones, L., Gomez, A. N., Kaiser, L., and Polosukhin, I. (2017). Attention is all you need. Preprint at arXiv. https://doi.org/10.48550/arXiv.1706.03762.
13. Pandey, R. (2024). gzip predicts data-dependent scaling laws. Preprint at arXiv. https://doi.org/10.48550/arXiv.2405.16684.
14. Hayes, T., Rao, R., Akin, H., Sofroniew, N.J., Oktay, D., Lin, Z., Verkuil, R., Tran, V.Q., Deaton, J., Wiggert, M., et al. (2024). Simulating 500 million years of evolution with a language model. Preprint at bioRxiv. https://doi.org/10.1101/2024.07.01.600583.
15. Lu, A. X. (2025). Code for cheap: Compressed hourglass embedding adaptation of proteins. Zenodo. https://doi.org/10.5281/zenodo.15128472. doi:.
16. Lu, A. X. (2025). Model weights for cheap: Compressed hourglass embedding adaptation of proteins. Hugging Face. https://doi.org/10.57967/hf/5067. doi.
17. Sun, M., Chen, X., Kolter, J.Z., and Liu, Z. (2024). Massive activations in large language models. Preprint at arXiv. https://doi.org/10.48550/arXiv.2402.17762.
18. Dettmers, T., Lewis, M., Belkada, Y., and Zettlemoyer, L. (2022). Gpt3. int8 : 8-bit matrix multiplication for transformers at scale. Adv. Neural Inf. Process. Syst. *35*, 30318–30332.
19. Rao, R.M., Liu, J., Verkuil, R., Meier, J., Canny, J., Abbeel, P., Sercu, T., and Rives, A. (2021). MSA Transformer. In Proceedings of the 38th International Conference on Machine Learning, *139*, pp. 8844–8856.
20. MacKay, D.J. (2003). Information Theory, Inference and Learning Algorithms (Cambridge university press).
21. He, K., Zhang, X., Ren, S., and Sun, J. (2016). Deep residual learning for image recognition. In Proceedings of the IEEE conference on computer vision and pattern recognition, pp. 770–778.
22. Nawrot, P., Tworkowski, S., Tyrolski, M., Kaiser, Ł., Wu, Y., Szegedy, C., and Michalewski, H. (2021). Hierarchical transformers are more efficient language models. Preprint at arXiv. https://doi.org/10.48550/arXiv.2110.13711.
23. Rombach, R., Blattmann, A., Lorenz, D., Esser, P., and Ommer, B. (2022). High-resolution image synthesis with latent diffusion models. In Proceedings of the IEEE/CVF Conference on Computer Vision and Pattern Recognition, pp. 10684–10695.
24. Ho, J., Jain, A., and Abbeel, P. (2020). Denoising diffusion probabilistic models. Adv. Neural Inf. Process. Syst. *33*, 6840–6851.
25. Song, J., Meng, C., and Ermon, S. (2020). Denoising diffusion implicit models. Preprint at arXiv. https://doi.org/10.48550/arXiv.2010.02502.
26. Esser, P., Rombach, R., and Ommer, B. (2021). Taming transformers for high-resolution image synthesis. In Proceedings of the IEEE/CVF conference on computer vision and pattern recognition, pp. 12873–12883.
27. Chang, H., Zhang, H., Jiang, L., Liu, C., and Freeman, W.T. (2022). Maskgit: Masked generative image transformer. In Proceedings of the IEEE/CVF Conference on Computer Vision and Pattern Recognition, pp. 11315–11325.
28. He, K., Chen, X., Xie, S., Li, Y., Dollár, P., and Girshick, R. (2022). Masked autoencoders are scalable vision learners. In Proceedings of the IEEE/CVF conference on computer vision and pattern recognition, pp. 16000–16009.
29. Razavi, A., Van den Oord, A., and Vinyals, O. (2019). Generating diverse high-fidelity images with vq-vae-2. Adv. Neural Inform. Process. Syst. *32*.
30. Sillitoe, I., Bordin, N., Dawson, N., Waman, V.P., Ashford, P., Scholes, H. M., Pang, C.S.M., Woodridge, L., Rauer, C., Sen, N., et al. (2021). Cath: increased structural coverage of functional space. Nucleic Acids Res. *49*, D266–D273.
31. Mistry, J., Chuguransky, S., Williams, L., Qureshi, M., Salazar, G.A., Sonnhammer, E.L.L., Tosatto, S.C.E., Paladin, L., Raj, S., Richardson, L. J., et al. (2021). Pfam: The protein families database in 2021. Nucleic Acids Res. *49*, D412–D419.
32. van Kempen, M., Kim, S.S., Tumescheit, C., Mirdita, M., Gilchrist, C.L., Söding, J., and Steinegger, M. (2022). Foldseek: fast and accurate protein structure search. Preprint at bioRxiv. https://doi.org/10.1101/2022.02.07.479398.
33. Gao, Z., Tan, C., and Li, S.Z. (2024). Foldtoken2: Learning compact, invariant and generative protein structure language. Preprint at bioRxiv. https://doi.org/10.1101/2024.06.11.598584.
34. Li, M., Tan, Y., Ma, X., Zhong, B., Yu, H., Zhou, Z., Ouyang, W., Zhou, B., Hong, L., and Tan, P. (2024). Prosst: Protein language modeling with quantized structure and disentangled attention. Preprint at bioRxiv. https://doi.org/10.1101/2024.04.15.589672.
35. Gaujac, B., Donà, J., Copoiu, L., Atkinson, T., Pierrot, T., and Barrett, T.D. (2024). Learning the language of protein structure. Preprint at arXiv. https://doi.org/10.48550/arXiv.2405.15840.
36. Van Den Oord, A., and Vinyals, O. (2017). Neural discrete representation learning. Adv. Neural Inf. Process. Syst. *30*. https://doi.org/10.48550/arXiv.1711.00937.
37. Takida, Y., Shibuya, T., Liao, W., Lai, C.-H., Ohmura, J., Uesaka, T., Murata, N., Takahashi, S., Kumakura, T., and Mitsufuji, Y. (2022). Sq-vae: Variational bayes on discrete representation with self-annealed stochastic quantization. Preprint at arXiv. https://doi.org/10.48550/arXiv.2205.07547.
38. Łańcucki, A., Chorowski, J., Sanchez, G., Marxer, R., Chen, N., Dolfing, H. J., Khurana, S., Alumäe, T., and Laurent, A. (2020). Robust training of vector quantized bottleneck models. In 2020 International Joint Conference on Neural Networks (IJCNN) (IEEE), pp. 1–7.
39. Dhariwal, P., Jun, H., Payne, C., Kim, J.W., Radford, A., and Sutskever, I. (2020). Jukebox: A generative model for music. Preprint at arXiv. https://doi.org/10.48550/arXiv.2005.00341.

40. Huh, M., Cheung, B., Agrawal, P., and Isola, P. (2023). Straightening out the straight-through estimator: Overcoming optimization challenges in vector quantized networks. In International Conference on Machine Learning (PMLR), pp. 14096–14113.
41. Mentzer, F., Minnen, D., Agustsson, E., and Tschannen, M. (2023). Finite scalar quantization: Vq-vae made simple. Preprint at arXiv. https://doi.org/10.48550/arXiv.2309.15505.
42. Xu, M., Zhang, Z., Lu, J., Zhu, Z., Zhang, Y., Chang, M., Liu, R., and Tang, J. (2022). Peer: a comprehensive and multi-task benchmark for protein sequence understanding. Adv. Neural Inf. Process. Syst. *35*, 35156–35173.
43. Li, F.-Z., Amini, A.P., Yue, Y., Yang, K.K., and Lu, A.X. (2024). Feature reuse and scaling: Understanding transfer learning with protein language models. Preprint at bioRxiv. https://doi.org/10.1101/2024.02.05.578959.
44. Romero, P.A., and Arnold, F.H. (2009). Exploring protein fitness landscapes by directed evolution. Nat. Rev. Mol. Cell Biol. *10*, 866–876.
45. Saharia, C., Chan, W., Saxena, S., Li, L., Whang, J., Denton, E.L., Ghasemipour, K., Gontijo Lopes, R., Karagol Ayan, B., Salimans, T., et al. (2022). Photorealistic text-to-image diffusion models with deep language understanding. Adv. Neural Inf. Process. Syst. *35*, 36479–36494.
46. Hesslow, D., Zanichelli, N., Notin, P., Poli, I., and Marks, D. (2022). RITA: a study on scaling up generative protein sequence models. Preprint at arXiv. https://doi.org/10.48550/arXiv.2205.05789.
47. Bengio, Y., Léonard, N., and Courville, A. (2013). Estimating or propagating gradients through stochastic neurons for conditional computation. Preprint at arXiv. https://doi.org/10.48550/arXiv.1308.3432.

Patterns, Volume *6*

Supplemental information

Tokenized and continuous embedding compressions of protein sequence and structure

Amy X. Lu, Wilson Yan, Kevin K. Yang, Vladimir Gligorijevic, Kyunghyun Cho, Pieter Abbeel, Richard Bonneau, and Nathan C. Frey

Tokenized and Continuous Embedding Compressions of Protein Sequence and Structure
Amy X. Lu[1,2,*,**], Wilson Yan[1], Kevin K. Yang[3], Vladimir Gligorijevic[2], Kyunghyun Cho[2,4], Pieter Abbeel[1], Richard Bonneau[2], and Nathan C. Frey[3]

[1]University of California, Berkeley
[2]Prescient Design, Genentech
[3]Microsoft Research
[4]New York University
[*]Correspondence: amyxlu@berkeley.edu
[**]Lead Contact: amyxlu@berkeley.edu

Supplemental information

Data S1. Code snippet for obtaining the embedding space used in this work.

Table S1. FSQ codebook levels used to obtain the implicit codebook size in Figure 4.

Table S2. Compression results on sequence-only datasets.

Table S3. Benchmarks on function and localization.

Table S4. Benchmarks on structural and contact tasks.

Figure S1. TSNE of embeddings after and before compression, colored by the "Architecture" level of the CATH classification hierarchy.

Figure S2. Exploring effects of corrupting the token representations on the structure space.

Extracting Embedding from ESMFold

We provide a code sketch of how to obtain the embedding space used in this work, based on modified and annotated source code from ESMFold[1]. Note that `self.esm_s_combine` and `self.esm_s_mlp` are both trained end-to-end with loss objectives from the original ESMFold paper; these weights add more structural awareness to the embedding space which we examine in this work than the original ESM2 3B embedding.

```
# self.esm_s_combine is a learnable weight
self.esm_s_combine = nn.Parameter(torch.zeros(self.esm.num_layers + 1))

# Forward pass through ESM2 language model.
# ‘esm_s‘ is a stack of representations from all layer outputs
# esm_s = torch.stack(
#   [v for _, v in sorted(res["representations"].items())], dim=2
# )
# Typically, when working with ESM2 LM embeddings, one would use
# embeddings from ‘res["representations"][final_layer_number]
esm_s, esm_z = self._compute_language_model_representations(esmaa)

# weigh representations from different layers
esm_s = (self.esm_s_combine.softmax(0).unsqueeze(0) @ esm_s).squeeze(2)

# Simple MLP for manipulating output representation
s_s_0 = self.esm_s_mlp(esm_s)

# Pairwise input is initialized to zero:
s_z_0 = s_s_0.new_zeros(B, L, L, self.cfg.trunk.pairwise_state_dim)

# Pointwise add embedding of amino acid
s_s_0 += self.embedding(aa)

########
# Use this representation for CHEAP experiments:
# return s_s_0
########

# In ESMFold code, this embedding is then used as input
# to the structure trunk. We also use this to
# reconstruct structure from the CHEAP embedding.

structure: dict = self.trunk(
    s_s_0, s_z_0, aa, residx, mask, no_recycles=num_recycles
)
```

Data S1: **Code snippet for obtaining CHEAP embeddings from ESMFold.** Adapted code snippet specifying the layer used to define the ESMFold latent space. We use the layer just prior to the structure module input, due to our observation that the pairwise input into the Structure Module is initialized as zeros (Main Text Figure 1).

FSQ codebook

To obtain the codebook sizes used in Figure 4, we use the same FSQ levels as in the original FSQ paper[2].

**Target Size** $\|C\|$	2^8	2^{10}	2^{12}	2^{14}	2^{16}
**FSQ levels**	[8, 6, 5]	[8, 5, 5, 5]	[7, 5, 5, 5, 5]	[8, 8, 8, 6, 5]	[8, 8, 8, 5, 5, 5]

Table S1: **FSQ levels used to define codebook sizes.** Mirroring the original FSQ paper, we use the same FSQ levels to define the codebook size $|C|$ in Figure 4.

Compression Results on Sequence-Only Datasets

	Method	Dimensions		Structure metrics				Sequence metrics
		Codebook	Dim.	TMScore		RMSD		Sequence recovery
				Mean	Median	Mean	Median	Mean
	Cont.	-	256 x 32	0.939	0.983	1.740	0.740	0.755
	Cont.	-	256 x 64	0.959	0.991	1.060	0.601	0.756
	Cont.	-	512 x 8	0.883	0.961	2.707	1.424	0.753
Pfam	FSQ	[8,8,8,6,5]	-	0.711	0.716	5.523	4.742	0.555
Pfam	FSQ	[16,16,16,16]	-	0.706	0.698	5.673	4.673	0.538
Pfam	Cont.	-	128 x 8	0.760	0.819	5.273	3.739	0.622
Pfam	Cont.		256 x 4	0.564	0.539	10.203	9.927	0.471
Pfam	Cont.	-	256 x 8	0.728	0.786	6.062	4.320	0.471
Pfam	Cont.	-	256 x 32	0.826	0.947	3.740	1.561	0.812
Pfam	Cont.	-	256 x 64	0.972	0.975	1.855	0.837	0.975
Pfam	Cont.		512 x 4	0.552	0.522	10.264	10.065	0.438
Pfam	Cont.	-	512 x 8	0.850	0.925	3.274	1.963	0.960
CATH	FSQ	[8,8,8,6,5]	-	0.853	0.861	3.670	3.668	0.820
CATH	FSQ	[16,16,16,16]	-	0.875	0.909	3.208	2.900	0.937
CATH	Cont.	-	256 x 8	0.856	0.918	4.170	2.647	0.991
CATH	Cont.	-	256 x 32	0.931	0.971	2.075	1.185	1.000
CATH	Cont.	-	256 x 64	0.928	0.992	1.769	0.489	1.000

Table S2: **Compression results on sequence-only datasets.** TMScore is calculated with respect to the ESMFold predicted structure, rather than the ground truth structure.

Comparison with Other Embedding Methods on PEER Benchmark

Extending Figure 5, we also include comparisons to other reported performances in Xu et al.[3]:

	# Dimensions	Flu ↑	Sta ↑	β-lac ↑	Sol ↑	Sub ↑	Bin ↑
DDE	400	0.64	0.65	**0.62**	0.60	0.49	0.77
Moran	240	0.40	0.32	0.38	0.58	0.31	0.56
LSTM	640	0.49	0.53	0.14	0.70	0.63	0.88
Transformer	512	0.64	0.65	0.26	0.70	0.56	0.76
CNN	21	**0.68**	0.64	0.78	0.64	0.59	0.83
ResNet	512	0.64	0.13	0.15	0.67	0.52	0.79
ProtBert	1024	0.34	0.70	**0.62**	0.59	0.59	0.82
ESM-1b	1280	0.43	**0.75**	0.53	0.67	**0.80**	0.92
**CHEAP (ours)**	4	0.14	0.40	0.13	0.60	0.33	0.68
	8	0.22	0.44	0.17	0.64	0.45	0.74
	16	0.27	0.55	0.23	0.65	0.54	0.84
	32	0.28	0.56	0.28	0.67	0.57	0.87
	64	0.31	0.56	0.28	0.69	0.62	0.90
	128	0.41	0.58	0.38	0.70	0.68	0.90
	256	0.47	0.60	0.41	0.71	0.72	0.92
	512	0.51	0.63	0.36	**0.72**	0.74	0.93
No compression	1024	0.52	0.64	0.45	**0.72**	0.76	**0.94**

Table S3: **Benchmarks on function and localization.** Full results for Main Text Figure 6 on the PEER benchmark[3], with baseline results directly taken from the PEER paper.

	# Dimensions	Cont	Fold	SSP	Yst
DDE	400	–	0.10	–	0.56
Moran	240	–	0.07	–	0.53
LSTM	640	0.26	0.08	0.69	0.54
Transformer	512	0.18	0.09	0.60	0.54
CNN	21	0.10	0.11	0.66	0.55
ResNet	512	0.20	0.09	0.70	0.49
ProtBert	1024	0.40	0.11	0.82	0.54
ESM-1b	1280	0.46	0.30	0.83	**0.66**
**CHEAP (ours)**	8	0.28	0.15	0.82	0.45
	64	**0.42**	0.45	0.85	0.48
	128	0.38	0.47	0.85	0.51
	256	0.23	0.50	0.85	0.51
	512	0.37	**0.53**	**0.86**	0.46

Table S4: **Benchmarks on structural and contact tasks.** Full results for Main Text Figure 6 on the PEER benchmark, with baseline results directly taken from the PEER paper.

Compressed Representation TSNE Plots

TSNE of embeddings after and before compression, colored by the "Architecture" level of the CATH classification hierarchy. Qualitative examination shows that despite $128\times$ compression, fold and topology information is still large preserved.

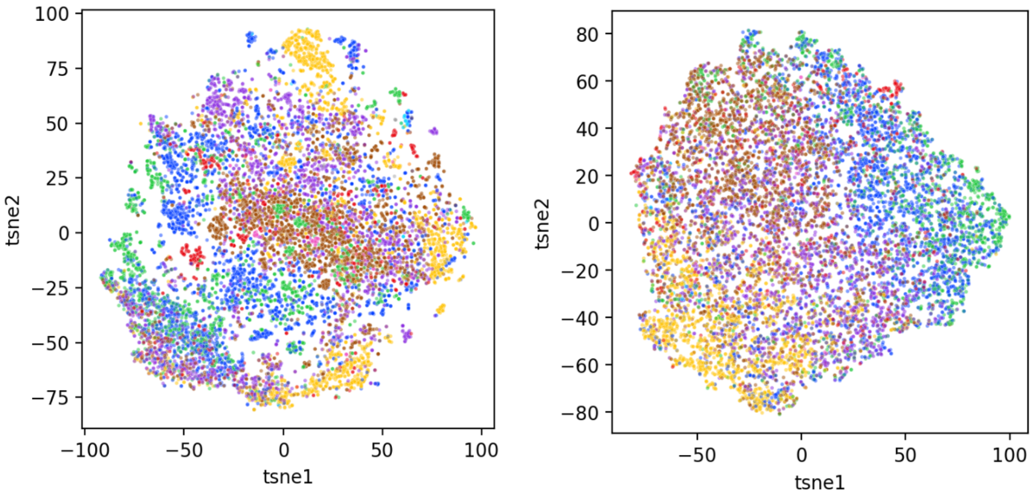

Figure S1: **TSNE of embeddings after and before compression.** Colored by the "Architecture" level of the CATH classification hierarchy.

Token Masking on Structure Reconstruction

Mirroring the analysis in Figure 7, we also examine the effect of "disturbing" the tokens on the output structure (Figure 4C), as a sanity check of how tokens encode structural information. The examined model uses FSQ levels $[16, 16, 16, 16]$, and has an implied codebook size of $C = 16^4 = 65536$. Since there is no `[MASK]` token in the vocabulary, we set different pre-quantization embeddings to 0. Generally, changing a subset of the tokens changes the structure, though since the codebook representations are not evenly spread through out, setting the first channels to zero has less of a detrimental effect.

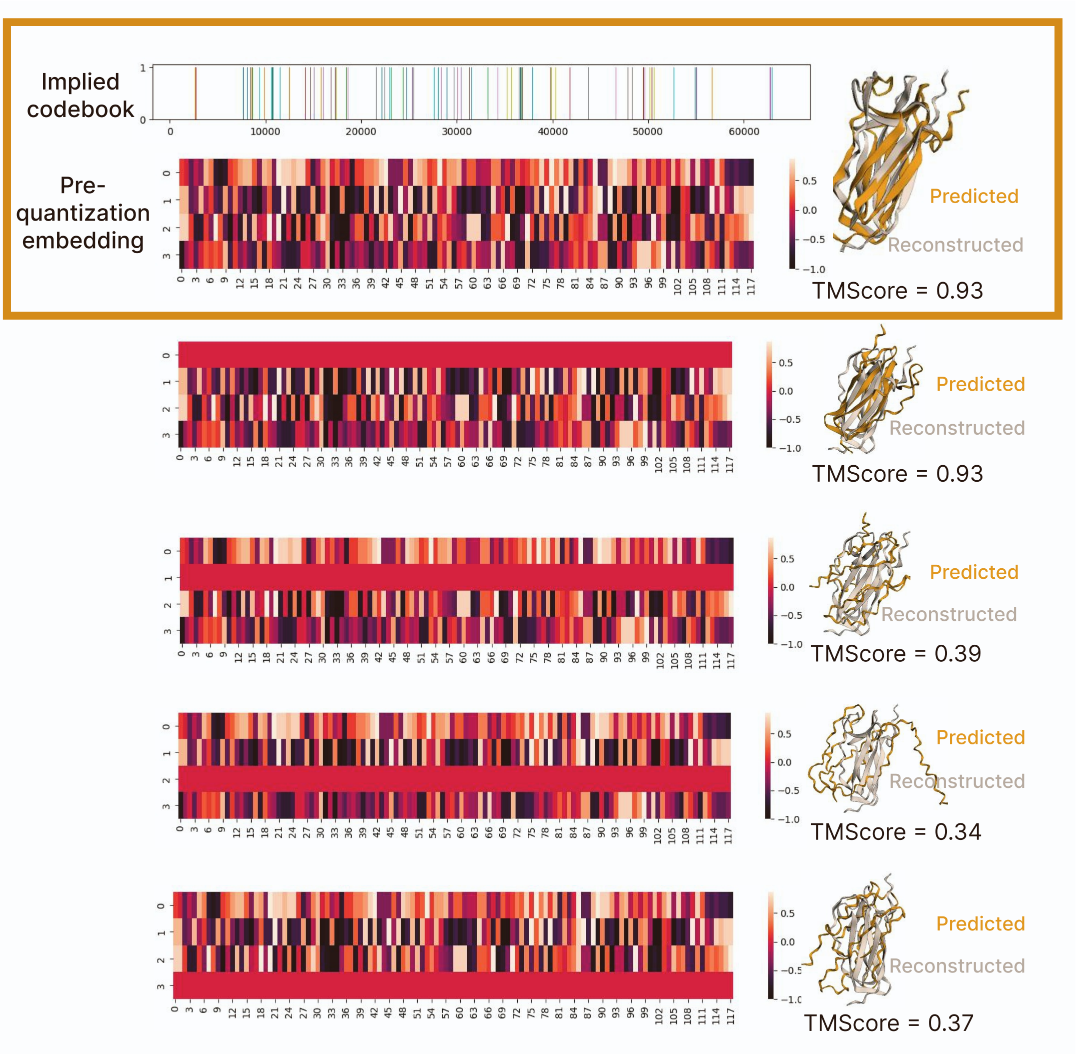

Figure S2: **Understanding token-space representation by corruption.** Exploring effects of token manipulation on the structure space for the $[16, 16, 16, 16]$ FSQ model, by setting some values to 0 just prior to quantization. The yellow box (top) denotes the original pre-quantization embedding and discrete codes representations of all-atom structure.

References

1. Lin, Z., Akin, H., Rao, R., Hie, B., Zhu, Z., Lu, W., Smetanin, N., Verkuil, R., Kabeli, O., Shmueli, Y. et al. (2023). Evolutionary-scale prediction of atomic-level protein structure with a language model. Science *379*, 1123–1130.

2. Mentzer, F., Minnen, D., Agustsson, E., and Tschannen, M. (2023). Finite scalar quantization: Vq-vae made simple. arXiv preprint arXiv:2309.15505.

3. Xu, M., Zhang, Z., Lu, J., Zhu, Z., Zhang, Y., Chang, M., Liu, R., and Tang, J. (2022). Peer: a comprehensive and multi-task benchmark for protein sequence understanding. Advances in Neural Information Processing Systems *35*, 35156–35173.
